# Low back pain prevalence and risk factors among health workers in Saudi Arabia: A systematic review and meta‐analysis

**DOI:** 10.1002/1348-9585.12155

**Published:** 2020-07-25

**Authors:** Hamad S. Al Amer

**Affiliations:** ^1^ Department of Physical Therapy Faculty of Applied Medical Sciences University of Tabuk Tabuk Saudi Arabia

**Keywords:** health personnel, low back pain, prevalence, risk factors, Saudi Arabia

## Abstract

**Objectives:**

Low back pain (LBP) has a major impact on health workers, and its prevalence and risk factors among them in Saudi Arabia have been investigated previously. However, the results have never been comprehensively reviewed. Therefore, the aim of this study was to perform a systematic review and meta‐analysis of the available literature to identify the prevalence and risk factors of LBP among health workers in Saudi Arabia.

**Methods:**

MEDLINE/PubMed, Web of Science, Scopus, CINAHL, and Saudi peer‐reviewed journals were searched for relevant literature. After quality assessment of the eligible articles, 18 studies targeting seven occupational categories, with a total number of 5345 health workers, were analyzed.

**Results:**

Pooled prevalence rates of 40.8% (n = 7 studies), 65.0% (n = 13 studies), and 81.4% (n = 2 studies) were obtained for week, year, and career, respectively, across all professional groups. Nurses and physical therapists were more susceptible to LBP, in that order, than the other categories considering week and career periods. Age, body mass index, and female gender were the most commonly reported individual risk factors. Occupational risk factors mainly included work‐related activities requiring back bending and twisting, lifting and pulling objects, and manual patient‐handling.

**Conclusions:**

The results of this review indicate that LBP is highly prevalent among health workers in Saudi Arabia when compared with international rates. Proper prophylactic measures are necessary to reduce LBP and minimize its consequences. Further high‐quality research is needed in different Saudi regions to achieve a broader understanding of LBP prevalence and causes.

## 1 INTRODUCTION

Low back pain (LBP) is highly prevalent around the world.^1^ In Saudi Arabia, its prevalence is estimated to range from 18.8%^2^ to 53.5%.^3^ At the same time, LBP is considered one of the leading reasons for loss of productive work time and missed workdays.^4^ In fact, 24.1% of workers in Saudi Arabia reported reduced working hours, 29.2% reported limited working activities, and 15.3% reported absence from work due to LBP.^3^

LBP is a common cause of absenteeism among health workers in Saudi Arabia.^5^, ^6^, ^7^, ^8^ Previous studies showed that 10.9%‐54.4% of health workers who had LBP reported taking sick days because of it.^8^, ^9^ For 71% of them, their sick leave may extend from 2 to 30 days.^6^ Around 70%‐85% believed that their LBP was caused by work‐related activities.^10^, ^11^, ^12^ In fact, 15%‐17% of health workers had to change their work setting because of LBP.^7^, ^8^ Other consequences of LBP reported by health workers in Saudi Arabia range from limited social, leisure, and daily activities^6^, ^7^, ^8^ to seeking medical help, hospital admission, and even surgery.^5^, ^6^, ^9^, ^13^, ^14^

In recent years, there has been a rapid increase in the number of published papers investigating the prevalence and risk factors of LBP across different categories of health workers in different parts of Saudi Arabia, with studies reporting a wide range of LBP prevalence rates. For instance, the annual prevalence of LBP across health workers in Saudi Arabia was estimated to range from 46.5%^15^ to 92.6%.^16^ These rates were attributed to various individual risk factors, such as age and gender. Work‐related factors were also reported, such as high workload, manual patient‐handling, and workplace. To the best of the author's knowledge, these studies have never been systematically analyzed.

### 1.1 Objectives and research questions

To achieve an overall understanding of the development of LBP and its associated risk factors in the healthcare sector in Saudi Arabia, the primary objectives of this review were (a) to estimate the prevalence of LBP among health workers in Saudi Arabia and (b) to identify the associated risk factors of LBP. The secondary objectives were to identify, when possible, the characteristics of LBP episodes in terms of duration and intensity, and compare the risk of developing LBP between the different occupational categories. Accordingly, the main research questions of this review were as follows: (a) What is the estimated prevalence of LBP among health workers in Saudi Arabia? and (b) What are the risk factors of LBP in this population?

## 2 MATERIALS AND METHODS

### 2.1 Search strategy

A search of the literature was conducted in the following electronic databases: MEDLINE/PubMed, Web of Science, Scopus, and CINAHL. The key terms used for performing the search were (“Saudi”) AND (“hospital” OR “physicians” OR “surgeons” OR “nurses” OR “dentists” OR “physical therapists” OR “clinicians" OR “health professionals” OR “health associate professionals” OR “healthcare workers” OR “healthcare professionals” OR “medical practitioners” OR “health personnel”) AND (“low back pain” OR “lower backache” OR “spinal pain” OR “spinal disorders” OR “musculoskeletal disorders” OR “musculoskeletal pain”) AND (“prevalence” OR “frequency” OR “incidence” OR “risk factors”). Furthermore, electronic Saudi peer‐reviewed journals were searched for relevant articles. Duplicate records were manually removed by the author. The abstracts of the obtained titles were examined for inclusion. If inclusion or exclusion could not be decided based on the abstract, the full text was retrieved to determine the eligibility of the study. The references of the retrieved articles were also inspected to identify additional potential publications. The author performed the literature search that extended until March 2020.

### 2.2 Inclusion and exclusion criteria

The obtained articles were screened by the author based on the following inclusion criteria: cross‐sectional full‐text articles published in a peer‐reviewed journal, conducted in Saudi Arabia, written in English, and investigating the prevalence and/or risk factors of LBP and/or musculoskeletal pain including LBP among health professionals and/or health associate professionals (according to the international classification of health workers of the World Health Organization),^17^ regardless of age or gender. Both classifications of LBP (specific and nonspecific) were considered. Excluded were review articles, letters to the editor, case reports, and editorials. Studies with an undefined prevalence period or including the general population or health students, interns, or cohorts other than health professionals and/or health associate professionals were also excluded.

### 2.3 Risk of bias and quality assessment

**FIGURE 1:**
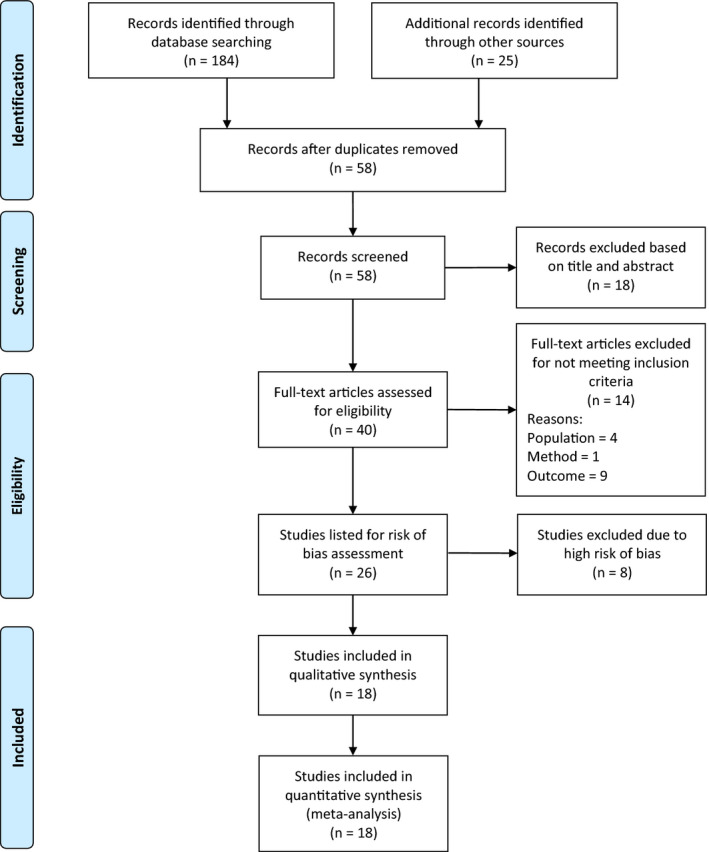
Flow diagram of the screening and selection process used in the review

All articles that fulfilled the eligibility criteria were assessed using the risk‐of‐bias tool developed by Hoy et al.^18^ This tool was designed mainly for prevalence studies and consists of 10 items addressing internal and external validity. Each item is scored as having either low or high risk of bias. If there was not sufficient information in the article to permit scoring a specific item, that item was scored as high risk of bias. The overall risk‐of‐bias score for each individual study was the total number of high‐risk items (considering a score of 0‐2 as low, 3‐4 as moderate, and 5‐10 as high risk of bias). Two independent raters performed the assessment of risk of bias, and the differences between the raters were resolved by discussion. To improve the quality of the results, studies with a high risk of bias were eventually excluded from the final analysis (Figure 1). A sensitivity analysis was performed to explore whether including studies with a high risk of bias affected the prevalence rates extracted only from studies with low and moderate risk of bias.

### 2.4 Data extraction

A data extraction form for prevalence and risk factor studies^19^ was adapted and modified to meet the purpose of this review. The form was used to extract the characteristics of the eligible studies, including study design, method, location, setting, occupational category, sample description, inclusion and exclusion criteria, outcome measure, prevalence rate, duration and intensity of LBP, and significant risk factors along with odds ratios (ORs) and 95% confidence intervals (95% CIs). For studies that reported risk factors for musculoskeletal disease in general, only the prevalence rates of LBP were extracted, as the risk factors were not specific for LBP. For the studies that did not report the number of cases, this was calculated based on the prevalence rate reported. Whenever essential data were missing from the article, or conflicts were noticed in the results, the authors were contacted for clarification or to obtain the missing information.

### 2.5 Statistical analysis and data synthesis

Separate meta‐analyses were conducted, and forest plots were generated, to obtain pooled prevalence rates and 95% CIs for each identified prevalence period and occupational category using a Microsoft Excel spreadsheet published by Neyeloff et al.^20^ The heterogeneity of the analyzed studies was examined using Cochran's Q and I^2^ statistics. The I^2^ values were interpreted as follows: <25%, low heterogeneity; 25%‐75%, moderate heterogeneity; and >75%, high heterogeneity.^21^ To calculate the pooled prevalence rates, either fixed‐effect or random‐effects models were used if heterogeneity was low or moderate to high, respectively.^20^

Furthermore, the risk of developing LBP for the identified occupational categories was estimated by calculating the ORs and 95% CIs. Chi‐square tests were used to examine the significance of the obtained ratios with an alpha level set at 0.05. All statistical tests were performed using IBM SPSS Statistics for Windows version 25.0 (Armonk, NY).

## 3 RESULTS

A total of 209 articles were obtained by searching the databases and references of the retrieved publications. After removing duplicates, 58 studies were screened, and 18 were excluded based on the title and abstract. The remaining 40 articles were identified as potentially relevant publications, and their full texts were retrieved and assessed for eligibility. A total of 14 studies were excluded for not meeting the inclusion criteria as follows: two studies included health students/interns; two included administration staff; four did not define the prevalence period; one was not fully conducted in Saudi Arabia; and five grouped LBP with upper back pain, defined as “back pain”. Finally, 26 articles^6^, ^7^, ^8^, ^9^, ^10^, ^13^, ^14^, ^15^, ^16^, ^22^, ^23^, ^24^, ^25^, ^26^, ^27^, ^28^, ^29^, ^30^, ^31^, ^32^, ^33^, ^34^, ^35^, ^36^, ^37^, ^38^ fulfilled the inclusion criteria and underwent a risk‐of‐bias assessment. Figure 1shows the Preferred Reporting Items for Systematic Reviews and Meta‐Analyses^39^ flow diagram illustrating the screening and selection process followed in the present review.

### 3.1 Risk of bias and quality assessment

For the assessment of risk of bias of the 26 articles, low risk of bias was found for 5 (19.2%) studies,^16^, ^24^, ^30^, ^32^, ^34^ moderate risk for 13 (50%),^6^, ^7^, ^9^, ^10^, ^13^, ^15^, ^23^, ^27^, ^28^, ^33^, ^35^, ^36^, ^37^ and high risk for 8 (30.8%).^8^, ^14^, ^22^, ^25^, ^26^, ^29^, ^31^, ^38^ The risk‐of‐bias assessment for the studies is shown in detail in Table 1. The sensitivity analysis revealed that excluding prevalence estimates of high risk‐of‐bias studies from the meta‐analyses did not substantially affect the prevalence rates, as will be shown later in this section.

**TABLE 1:** Risk‐of‐bias scores for included and excluded articles

Study	External validity				Internal validity						Overall
	Representation	Sampling	Random selection	Non‐response bias	Data collection	Case definition	Validity of instrument	Consistency of data collection	Prevalence period	Numerators and denominators	
*Included articles (*n* = 18)*											
Al‐Eisa and Al‐Abbad, 2013^6^	High	High	High	Low	Low	Low	Low	Low	Low	Low	Moderate
Alghadir et al, 2017^7^	High	High	High	Low	Low	Low	High	Low	Low	Low	Moderate
Abbas et al, 2010^9^	High	Low	Low	High	Low	Low	High	Low	Low	Low	Moderate
Alsiddiky et al, 2015^10^	High	Low	Low	Low	Low	High	Low	Low	High	Low	Moderate
Alzidani et al, 2018^13^	High	High	High	Low	Low	Low	High	Low	Low	Low	Moderate
Muaidi and Shanb, 2016^15^	Low	High	High	High	Low	High	Low	Low	Low	Low	Moderate
Alnaami et al, 2019^16^	High	Low	Low	Low	Low	Low	Low	Low	Low	Low	Low
Abu Tariah et al, 2020^23^	High	High	High	High	Low	Low	Low	Low	Low	Low	Moderate
Al Shammari et al, 2019^24^	High	Low	High	Low	Low	Low	Low	Low	Low	Low	Low
Aljanakh et al, 2015^27^	High	High	High	Low	Low	Low	Low	Low	Low	High	Moderate
Aljerian et al, 2018^28^	High	High	High	Low	Low	Low	Low	Low	Low	High	Moderate
Al‐Mohrej et al, 2016^30^	High	Low	Low	Low	Low	Low	Low	Low	Low	Low	Low
Al‐Ruwaili and Khalil, 2019^32^	High	Low	Low	Low	Low	High	Low	Low	Low	Low	Low
Alsultan et al, 2018^33^	High	High	High	Low	Low	Low	Low	Low	Low	Low	Moderate
Attar, 2014^34^	High	Low	Low	Low	Low	Low	Low	Low	Low	Low	Low
Bin Homaid et al, 2016^35^	High	Low	High	Low	Low	Low	Low	Low	High	Low	Moderate
Keriri, 2013^36^	High	High	Low	High	Low	Low	High	Low	Low	Low	Moderate
Mohamed and Al Amer, 2019^37^	High	High	High	Low	Low	Low	Low	Low	Low	High	Moderate
*Excluded articles (*n* = 8)*											
Al Dajah and Al Daghdi, 2013^8^	High	High	High	Low	Low	High	High	Low	Low	Low	High
Aseri et al, 2019^14^	High	Low	High	High	Low	High	Low	Low	High	Low	High
Abduljabbar, 2008^22^	High	High	Low	High	Low	High	High	Low	Low	High	High
Alamri et al, 2018^25^	High	Low	Low	High	Low	High	High	Low	Low	High	High
Alghadir et al, 2015^26^	High	High	High	High	Low	High	High	Low	High	High	High
Almalki et al, 2016^29^	High	Low	High	Low	Low	High	High	Low	High	Low	High
AlNekhilan et al, 2020^31^	High	High	High	High	Low	Low	Low	Low	Low	High	High
Gaowgzeh, 2019^38^	High	High	Low	Low	Low	High	High	Low	High	Low	High

### 3.2 Study characteristics

A total of 18 articles were included in the final analysis. All of them were cross‐sectional, and their characteristics are listed in Table 2. The study sample sizes ranged from 68^27^ to 937,^9^ with a total number of 5345 health workers. Out of the 18 studies included, 5 targeted physicians,^10^, ^13^, ^24^, ^32^, ^33^ 2 focused on dentists,^27^, ^30^ 5 studied nurses,^6^, ^9^, ^23^, ^34^, ^36^ 2 targeted physical therapists,^7^, ^15^ 1 studied emergency medical service (EMS) staff,^28^ and 3 included more than one category of health workers.^16^, ^36^, ^37^ Other health workers were anesthesia technicians (AT) and operation room technicians (ORT).^35^

**TABLE 2:** Characteristics of the studies included in the review

Study	Design	City	Setting	Period	Occupation	Sample size	Age (y)	Inclusion/exclusion criteria	Outcome measure	Definition of low back pain
Al‐Eisa and Al‐Abbad, 2013^6^	Cross‐sectional	Riyadh	1 hospital	Not reported	Nurses	155 M = 29 F = 126	39.8 ± 8.2	Inclusion: work full time in the rehabilitation hospital and responsible for patient handling activities Exclusion: pregnancy and health‐related problems prohibiting from handling patients	Self‐designed, four‐part questionnaire; part 4 is the Standardized Nordic Musculoskeletal Questionnaire	Defined by the Standardized Nordic Musculoskeletal Questionnaire
Alghadir et al, 2017^7^	Cross‐sectional	Riyadh	NA; online survey	Not reported	PTs	502 M = 307 F = 195	<30 to >40	Inclusion: in direct patient contact for at least 1 h each day	Self‐designed questionnaire	Unpleasant sensation in the lower back region below the scapulae and above the hip region, that may or may not radiate to the thighs and legs
Abbas et al, 2010^9^	Cross‐sectional	Riyadh	4 hospitals	Not reported	Nurses	937 M = 114 F = 823	<30 to >50	Exclusion: history of musculoskeletal or bone disorders	Self‐designed questionnaire and the Standardized Nordic Musculoskeletal Questionnaire	Defined by the Standardized Musculoskeletal Nordic Questionnaire
Alsiddiky et al, 2015^10^	Cross‐sectional	Riyadh	1 hospital	May 2013 to August 2013	Physicians	411 M = 248 F = 163	20‐50	Not reported	Self‐designed questionnaire	No definition reported
Alzidani et al, 2018^13^	Cross‐sectional	Taif	3 hospitals	January to March 2018	Physicians	138 M = 107 F = 31	≤ 30 to >50	Exclusion: history of back or spinal surgery, any fracture or disorder in the pelvic region, spinal deformities, osteoporosis, back or spinal tumor, or any other malignancies	Self‐designed questionnaire	Pain, muscle tension, or stiffness localized below the costal margin and above the inferior gluteal folds, with or without leg pain
Muaidi and Shanb, 2016^15^	Cross‐sectional	Nationwide	NA	Not reported	PTs	690 M = 408 F = 282	Not reported	Exclusion: less than 1 y in their current work settings or reported musculoskeletal pain as a result of previous trauma	Self‐designed questionnaire	No definition reported
Alnaami et al, 2019^16^	Cross‐sectional	Aseer	Hospitals and primary healthcare centers	Not reported	Physicians Dentists Nurses	594	20 to >50	Exclusion: retired or not practicing clinical work	Self‐designed questionnaire	Pain, muscle tension, or stiffness localized below the costal margin and above the inferior gluteal folds, with or without leg pain (sciatica)
Abu Tariah et al, 2020^23^	Cross‐sectional	Riyadh	1 hospital	Not reported	Nurses	94 M = 2 F = 92	<25 to ≥50	Inclusion: at least 1 y of work experience with direct patients' care Exclusion: not involved in direct patients' care such as nurse administrators and students	The Standardized Nordic Musculoskeletal Questionnaire	Defined by the Standardized Nordic Musculoskeletal Questionnaire
Al Shammari et al, 2019^24^	Cross‐sectional	Eastern Province	12 health institutions	April, 2019	Physicians	198 M = 111 F = 87	<30 to ≥50	Not reported	Self‐designed, four‐part questionnaire and the Standardized Nordic Musculoskeletal Questionnaire	Defined by the Standardized Nordic Musculoskeletal Questionnaire
Aljanakh et al, 2015^27^	Cross‐sectional	Ha'il	Governmental hospitals and clinics	January 2014 to January 2015	Dentists	68 M = 43 F = 25	38.5 ± 7.4	Inclusion: at least 1 y of work experience in the current position	Self‐designed questionnaire based on the Standardized Nordic Musculoskeletal Questionnaire	Defined by the Standardized Nordic Musculoskeletal Questionnaire
Aljerian et al, 2018^28^	Cross‐sectional	Riyadh	SRCA and hospitals	Not reported	EMS	360 all males	Not reported	Exclusion: dispatchers and non‐shift EMS personnel	Self‐designed, two‐part questionnaire; part 2 is the Standardized Nordic Musculoskeletal Questionnaire	Defined by the Standardized Nordic Musculoskeletal Questionnaire
Al‐Mohrej et al, 2016^30^	Cross‐sectional	Riyadh	150 hospitals and private clinics	Not reported	Dentists	204 M = 103 F = 101	38.0 ± 10.6	Inclusion: worked as a dentist for at least 1 y Exclusion: history of orthopedic trauma or congenital deformities (of the neck, back and upper extremities)	Self‐designed questionnaire based on the Standardized Nordic Musculoskeletal Questionnaire	Defined by the Standardized Nordic Musculoskeletal Questionnaire
Al‐Ruwaili and Khalil, 2019^32^	Cross‐sectional	Tabuk	1 hospital	2019	Physicians	254 M = 170 F = 84	36.0 ± 9.3	Inclusion: physicians, from both genders, all specialties and qualifications working during 2019 Exclusion: chronic or recurrent back pain, trauma in the back, osteoporosis, infection, or neoplasm	Self‐designed questionnaire	No definition reported
Alsultan et al, 2018^33^	Cross‐sectional	Riyadh	1 hospital	Not reported	Physicians	140 M = 110 F = 30	27	Not reported	Self‐designed, two‐part questionnaire; part 2 is the Standardized Nordic Musculoskeletal Questionnaire	Defined by the Standardized Nordic Musculoskeletal Questionnaire
Attar, 2014^34^	Cross‐sectional	Jeddah	1 hospital	September, 2011 to February, 2012	Nurses	200 M = 9 F = 191	34.6 ± 8.1	Exclusion: employees other than nurses	Self‐designed, three‐part questionnaire; part 3 is the Standardized Nordic Musculoskeletal Questionnaire	Defined by the Standardized Nordic Musculoskeletal Questionnaire: Symptoms (pain, numbness, tingling, aching, stiffness, and burning) that resulted from a work‐related event, excluding other injuries experienced over the past year that lasted 1 wk or more or occurred at least monthly with at least moderate pain on average
Bin Homaid et al, 2016^35^	Cross‐sectional	Makkah	1 hospital	June, 2014	Physicians Nurses AT ORT	114	33.9 ± 7.6	Not reported	Self‐designed questionnaire	Pain, muscle tension, or stiffness localized below the costal margin and above the inferior gluteal folds, with or without leg pain (sciatica)
Keriri, 2013^36^	Cross‐sectional	Taif	4 hospitals	January to June 2011	Nurses	126 M = 27 F = 99	34.0 ± 8.0	Inclusion: nurses from both genders, belonging to any ethnic group, age less than 60 y, and working in operating rooms Exclusion: nurses with specific causes of back pain as a result of trauma, osteoporotic fractures, infections, and neoplasms	Self‐designed questionnaire	Experiencing pain, ache, or discomfort in the lower back
Mohamed and Al Amer, 2019^37^	Cross‐sectional	Tabuk	6 hospitals and 4 clinics	Not reported	Physicians Nurses PTs	160 M = 66 F = 94	35.5 ± 12.4	Inclusion: in direct contact with patients, work in both government and private hospitals or polyclinics Exclusion: work as a part timer, pregnant or on leave from their duties	Cornell Musculoskeletal Discomfort Questionnaire for Male and Female and Self‐designed questionnaire	Defined by Cornell Musculoskeletal Discomfort Questionnaire

Abbreviations: AT, anesthesia technicians; EMS, emergency medical service personnel; F, female; M, male; NA, not applicable; ORT, operation room technicians; PTs, physical therapists; SRCA, Saudi Red Crescent Authority.

One study^16^ evaluated the LBP prevalence in paramedics, and another study^35^ examined the prevalence among central sterile supply department staff. The prevalence rates for those two categories were not extracted, as the former study did not provide a clear description of health workers under the paramedics category, and central sterile supply department staff in the latter study do not fall under either health professionals or health associate professionals.^17^ Furthermore, these two studies reported risk factors for more than one occupational category without segregation. One reported the risk factors for operation room staff including physicians, nurses, AT, ORT, and central sterile supply department staff combined.^35^ The other study reported risk factors for physicians, dentists, nurses, and paramedics.^16^ A decision was made to include the risk factors reported in the latter study, as the authors examined job title as a risk factor of LBP, and it was found not significant. Therefore, it is reasonable to assume that the reported risk factors can be correlated with the occupational categories included in the study, since there was no significant difference in LBP prevalence between them.

Intensity and/or duration of LBP episodes were reported in 9 out of the 18 studies (see Table 3). Duration was reported by two studies with nurses,^6^, ^36^ and one study each with physicians,^32^ dentists,^30^ and physical therapists.^7^ Intensity of LBP episodes was reported by seven studies using different methods. Four studies surveyed the intensity among physicians,^10^, ^13^ dentists,^30^ and physical therapists^7^ on an ordinal scale of mild, moderate, or severe pain. Another study^35^ used the same method to measure LBP intensity among more than one occupational category; however, it added “very severe” to the scale. Three studies measured intensity in physicians,^32^ nurses,^36^ and among different occupational categories^37^ using numerical rating scales.

**TABLE 3:** Duration and intensity of low back pain episodes

Occupation	Study	Duration	Intensity
Physicians	Alsiddiky et al, 2015^10^	Not reported	Mild = 83 (34) Moderate = 68 (28) Severe = 93 (38)
	Alzidani et al, 2018^13^	Not reported	Mild = 34 (33.7) Moderate = 60 (59.4) Severe = 7 (6.9)
	Al‐Ruwaili and Khalil, 2019^32^	Duration of last episode: 0‐<1 wk = 109 (56.5) 1‐2 wks = 32 (16.6) 3‐4 wks = 26 (13.5) 4‐5 wks = 13 (6.7) >5 wks = 13 (6.7)	Intensity during the past 3 mo on a scale of 0‐5: 0 = 12 (6.2) 1 = 25 (13.0) 2 = 62 (32.1) 3 = 70 (36.3) 4 = 18 (9.3) 5 = 6 (3.1)
Dentists	Al‐Mohrej et al, 2016^30^	<4 wks = 112 (80.6) 2‐3 mo = 19 (13.7) 3‐6 mo = 6 (4.3) >6 mo = 2 (1.4)	Mild = 14 (10.1) Moderate = 92 (66.2) Severe = 33 (23.8)
Nurses	Al‐Eisa and Al‐Abbad, 2013^6^	1‐7 d = 65 (56.0) 8‐30 d = 30 (25.9) >30 d = 9 (7.8) Every day = 12 (10.3)	Not reported
	Keriri, 2013^36^	<2 y = 31 (50.8) >2 y = 30 (49.2)	Intensity during the past 3 mo on a scale of 1‐5: 1 = 9 (14.8) 2 = 23 (37.7) 3 = 20 (32.8) 4 = 6 (9.8) 5 = 3 (4.9)
PTs	Alghadir et al, 2017^7^	<1 wk = 250 (55) 2‐4 wks = 135 (30) >4 wks = 65 (14)	Mild = 216 (43) Moderate = 126 (25) Severe = 108 (22)
Physicians Nurses AT ORT	Bin Homaid et al, 2016^35^	Not reported	Mild = 32 (36) Moderate = 48 (53.9) Severe = 7 (7.9) Very severe = 2 (2.2)
Physicians Nurses PTs	Mohamed and Al Amer, 2019^37^	Not reported	Average of intensity during the past wk on a scale of 0‐10: Physicians: 3.31 Nurses: 4.55 PTs: 3.75 Overall: 4.01

Note

Values of duration and intensity are given as number (percentage) unless otherwise indicated.

Abbreviations: AT, anesthesia technicians; ORT, operation room technicians; PTs, physical therapists.

The 18 studies were conducted in different cities in Saudi Arabia, with the majority (8 studies) in Riyadh,^6^, ^7^, ^9^, ^10^, ^23^, ^28^, ^30^, ^33^ 2 each in Taif^13^, ^36^ and Tabuk,^32^, ^37^ and 1 each in Eastern Province,^24^ Jeddah,^34^ Asser,^16^ Makkah,^35^ and Ha'il.^27^ One study was conducted nationwide.^15^

### 3.3 Outcome measures

All the 18 studies used self‐developed questionnaires to measure the prevalence and associated risk factors of LBP in their samples. However, nine studies^6^, ^9^, ^23^, ^24^, ^27^, ^28^, ^30^, ^33^, ^34^ incorporated the Standardized Nordic Musculoskeletal Questionnaire^40^ in their tools, while one study^37^ integrated the Cornell Musculoskeletal Discomfort Questionnaire.^41^

### 3.4 Prevalence of LBP

Six different prevalence periods were identified, namely point, week, month, year, career (defined as the incidence of LBP at some point during the professional career),^7^, ^35^ and lifetime prevalence, with some studies reporting more than one prevalence period. The most commonly reported prevalence period was year prevalence (13 studies), followed by week prevalence (4 studies), point and lifetime prevalence (3 studies each), career prevalence (2 studies), and month prevalence (1 study). In this review, episodes occurring in the past 7 days or less (ie, week and point prevalence) were pooled together as week prevalence.^42^

It should be noted that one study^9^ calculated the lifetime prevalence exclusive of point and previous year episodes, unlike the other studies included. To ensure consistency across the studies reporting lifetime prevalence, only point and year prevalence rates were extracted from this study, and the lifetime prevalence was excluded. Additionally, one study^35^ calculated the prevalence for two medicine specialties separately (surgery and anesthesiology). Since the aim of this review was to study the prevalence among health workers regardless of the specialty within their field, the prevalence rates of the two specialties were combined under physicians.

#### 3.4.1 Prevalence and odds ratios of LBP by period

Pooled rates of 40.8% (95% CI = 28.4%‐53.2%; n = 7 studies), 65.0% (95% CI = 59.4%‐70.5%; n = 13 studies), and 81.4% (95% CI = 69.3%‐93.5%; n = 2 studies) were obtained for week, year, and career, respectively, across all professional groups (Table 4; Figures S1‐S3). Month and lifetime prevalence rates were identified only for physicians and were therefore described in the following section.

**TABLE 4:** Prevalence rates and odds ratios of low back pain by period and by occupational category

Prevalence period	Occupation	Study	No. of cases	Sample size	Prevalence		Online supporting information (related forest plot)	Risk of LBP		
					%	95% CI		OR	95% CI	*P* value
Week	Physicians	Alzidani et al, 2018^13^	28	138	20.3	12.8‐27.8				
		Al Shammari et al, 2019^24^	84	198	42.4	33.4‐51.5				
		Mohamed and Al Amer, 2019^37^	26	52	50.0	30.8‐69.2				
		*Pooled prevalence*			*36.4*	*18.0‐54.7*	Figure S4	Reference group		
	Nurses	Abbas et al, 2010^9^	576	937	61.5	56.5‐66.5				
		Abu Tariah et al, 2020^23^	16	94	17	8.7‐25.4				
		Keriri, 2013^36^	61	126	48.4	36.3‐60.6				
		Mohamed and Al Amer, 2019^37^	46	80	57.5	40.9‐74.1				
		*Pooled prevalence*			*45.9*	*21.9‐69.9*	Figure S8	2.35^a^	1.86‐3.00	<.0005
	PTs	Mohamed and Al Amer, 2019^37^	10	28	35.7	13.6‐57.9		1.01	0.45‐2.24	.987
	EMS	Aljerian et al, 2018^28^	134	360	37.2	30.9‐43.5		1.07	0.79‐1.44	.638
	*Pooled prevalence*				*40.8*	*28.4‐53.2*	Figure S1			
Month	Physicians	Alzidani et al, 2018^13^	67	138	48.6	41.0‐56.1				
Year	Physicians	Alzidani et al, 2018^13^	91	138	65.9	52.4‐79.5				
		Al Shammari et al, 2019^24^	137	198	69.2	57.6‐80.8				
		Alnaami et al, 2019^16^	259	353	73.4	64.4‐82.3				
		Al‐Ruwaili and Khalil, 2019^32^	177	254	69.6	59.4‐80.0				
		Alsultan et al, 2018^33^	74	140	52.9	40.8‐64.9				
		*Pooled prevalence*			*66.8*	*59.9‐73.7*	Figure S5	Reference group		
	Dentists	Aljanakh et al, 2015^27^	39	68	57.4	39.4‐75.4				
		Al‐Mohrej et al, 2016^30^	139	204	68.1	56.8‐79.5				
		Alnaami et al, 2019^16^	25	27	92.6	56.3‐128.3				
		*Pooled prevalence*			*67.3*	*54.1‐80.5*	Figure S7	0.98	0.75‐1.30	.934
	Nurses	Al‐Eisa and Al‐Abbad, 2013^6^	116	155	74.8	61.2‐88.5				
		Abbas et al, 2010^9^	611	937	65.2	60.0‐70.4				
		Abu Tariah et al, 2020^23^	60	94	63.8	47.7‐80.0				
		Alnaami et al, 2019^16^	156	214	72.9	61.5‐84.3				
		Attar, 2014^34^	130	200	65.0	53.8‐76.2				
		*Pooled prevalence*			*66.9*	*62.9‐70.9*	Figure S9	0.95	0.80‐1.12	.557
	PTs	Muaidi and Shanb, 2016^15^	321	690	46.5	41.4‐51.6		0.40^a^	0.33‐0.49	<.0005
	EMS	Aljerian et al, 2018^28^	217	360	60.3	52.3‐68.3		0.71^a^	0.55‐0.91	.006
	*Pooled prevalence*				*65.0*	*59.4‐70.5*	Figure S2			
Career	Physicians	Bin Homaid et al, 2016^35^	45	61	73.8	52.2‐95.3		Reference group		
	Nurses	Bin Homaid et al, 2016^35^	26	34	76.5	47.1‐105.9		1.15	0.43‐3.06	.772
	PTs	Alghadir et al, 2017^7^	450	502	89.6	81.4‐97.9		3.01^a^	1.62‐5.82	<.0005
	AT	Bin Homaid et al, 2016^35^	10	12	83.3	31.7‐135.0		1.78	0.35‐9.00	.482
	ORT	Bin Homaid et al, 2016^35^	3	7	42.9	−5.6 to 91.4		0.27	0.05‐1.32	.089
	*Pooled prevalence*				*81.4*	*69.3‐93.5*	Figure S3			
Lifetime	Physicians	Alsiddiky et al, 2015^10^	244	411	59.4	51.9‐66.8				
		Alzidani et al, 2018^13^	101	138	73.2	58.9‐87.5				
		Al‐Ruwaili and Khalil, 2019^32^	193	254	76.0	65.3‐86.7				
		*Pooled prevalence*			*68.7*	*57.0‐80.4*	Figure S6			

Abbreviations: AT, anesthesia technicians; CI, confidence interval; EMS, emergency medical service personnel; LBP, low back pain; OR, odds ratio; PTs, physical therapists; RT, operation room technicians.

^a^ Significant at α = 0.05.

Taking physicians as the reference group, Table 4 shows the ORs of developing LBP for each of the identified category for week, year, and career prevalence periods. For week prevalence, nurses were more likely to develop LBP with a significant OR of 2.35 (95% CI = 1.86‐3.00). For year prevalence, the risk was similar for dentists and nurses with no significant difference. Physical therapists and EMS personnel, however, had a significantly lower risk of LBP than physicians, with ORs of 0.40 (95% CI = 0.33‐0.49) and 0.71 (95% CI = 0.55‐0.91), respectively. For career prevalence, physical therapists had the highest risk of developing LBP, with a significant OR of 3.01 (95% CI = 1.62‐5.82).

#### 3.4.2 Prevalence of LBP by occupational category

Detailed prevalence rates for each occupational category are listed in Table 4 and described below.

##### Physicians

The week prevalence of LBP among physicians was reported in three studies,^13^, ^24^, ^37^ with a pooled prevalence of 36.4% (Figure S4). Only one study reported month prevalence, and another study reported career prevalence, which were 48.6%^13^ and 73.8%,^35^ respectively. Year prevalence was reported in five studies,^13^, ^16^, ^24^, ^32^, ^33^ ranging from 52.9%^33^ to 73.4%,^16^ with a pooled prevalence of 66.8% (Figure S5). Finally, three studies^10^, ^13^, ^32^ reported lifetime prevalence, with a pooled prevalence of 68.7% (Figure S6).

##### Dentists

For dentists, only the year prevalence of LBP was identified in the analysis and was reported in three studies,^16^, ^27^, ^30^ ranging between 57.4%^27^ and 92.6%,^16^ with a pooled prevalence of 67.3% (Figure S7).

##### Nurses

The week prevalence of LBP among nurses was reported in four studies,^9^, ^23^, ^36^, ^37^ ranging from 17.0%^23^ to 61.5%,^9^ with a pooled prevalence of 45.9% (Figure S8). The year prevalence was reported in five studies^6^, ^9^, ^16^, ^23^, ^34^ and ranged between 63.8%^23^ and 74.8%,^6^ with a pooled prevalence of 66.9% (Figure S9). The career prevalence of LBP was reported in one study only (76.5%).^35^

##### Physical therapists

Week, year, and career prevalence rates among physical therapists were reported in one study each, and were 35.7%,^37^ 46.5%,^15^ and 89.6%,^7^ respectively.

##### EMS, AT, and ORT

Only one study^28^ reported the prevalence of LBP for EMS, estimating the week and year prevalence to be 37.2% and 60.3%, respectively. For AT and ORT, only their career prevalence was reported in one study (83.3% and 42.9%, respectively).^35^

### 3.5 Duration and intensity of LBP episodes

Table 3 summarizes the information about the duration and/or intensity of LBP episodes that were reported in the studies included in the final analysis. The majority of physicians (86.6%),^32^ dentists (80.6%),^30^ and physical therapists (85%)^7^ described their LBP as acute (less than 4 weeks). For nurses, one study^6^ reported that 81.9% of their episodes had lasted for 30 days or less, while another study^36^ stated that approximately half of the sample had LBP for more than 2 years and the other half for less than 2 years.

Regarding the intensity of LBP episodes, one study^13^ stated that the majority of physicians (59.4%) described their LBP episodes as moderate; while another study^10^ reported that around 38% had severe LBP, slightly higher than those who described their pain as mild (34%). One study^32^ reported that 68.4% of the physicians rated their episodes as 2 or 3 on a scale of 0‐5. As for dentists, around 66.2% reported moderate levels of LBP.^30^ Similarly, most physical therapists (43%) rated their LBP as moderate.^7^ On a scale of 1‐5, approximately 70.5% of nurses selected either 2 or 3 to describe their LBP intensity.^36^ One study^35^ recorded LBP intensity among samples of physicians, nurses, AT, and ORT, and reported that 53.9% described their pain as moderate. Another study^37^ found that the overall average of pain, on a scale of 0‐10, among a group of physicians, nurses, and physical therapist was 4.01.

### 3.6 Risk factors for LBP

Several statistically significant risk factors for LBP among health workers across different prevalence periods were identified in the included studies. The risk factors along with ORs and 95% CIs for each occupational category are shown in Table 5, and were classified under two categories: individual and occupational risk factors. Overall, the most frequently reported individual factors were age,^10^, ^16^, ^30^ body mass index (BMI),^9^, ^16^, ^33^ and gender.^9^, ^10^, ^15^, ^30^, ^36^ As for occupational risk factors, type of work activities,^6^, ^9^, ^10^, ^30^ work setting,^13^, ^16^, ^34^ and specialty^10^, ^13^, ^30^, ^32^ were the factors most commonly found to be significant.

**TABLE 5:** Individual and occupational risk factors identified for each occupational category

Occupation	Prevalence period	Risk factor		Reference group	OR	95% CI	*P* value	Reference number
Physicians	Week	No risk factors identified						
	Month	No risk factors identified						
	Year	Individual	Age: 30 to <40 y	20 to <30	1.87	1.26‐2.75	<.05	16
			History of back trauma	No history of back trauma	10.44 11.52^a^	3.79‐28.78 4.14‐32.08^a^	<.05 <.05^a^	16
			BMI: obesity	Normal weight	1.72 1.10^a^	1.04‐2.83 1.01‐3.65^a^	<.05 <.05^a^	16
			BMI: obesity	Not reported			.04	33
		Occupational	Workplace: secondary and tertiary hospitals	Primary	1.80 1.32^a^	1.25‐2.59 1.01‐1.76^a^	<.05 <.05^a^	16
			Prolonged standing working conditions	Prolonged sitting	1.61	1.01‐2.56	<.05	16
			Specialty: ophthalmology, emergency, anesthesia and intensive care	Not reported			.014	32
	Career	No risk factors identified						
	Lifetime	Individual	Age: 31‐40 y	<30	2.2	1.1‐4.6	.004	10
			Age: 41 to >50 y	<30	3.0	1.4‐2.2	.004	10
			Smoking	Not reported			.033	13
			Nationality: non‐Saudi	Not reported			.02	13
			Male gender	Females	1.7^a^	1.1‐2.8^a^	.033^a^	10
		Occupational	Job position: consultant	Resident	2.5 4.1^a^	1.5‐4.2 2.1‐8.3^a^	.002 <.001^a^	10
			Job position: registrar	Resident	2.2^a^	1.2‐4.2^a^	.013^a^	10
			Specialty: surgery	Medicine	2.0	1.3‐3.0	.001	10
			Specialty: pediatrics	Medicine	2.4	1.3‐4.3	.001	10
			Specialty: orthopedic	Not reported			.012	13
			Specialty: gynecology	Not reported			.012	13
			Specialty: general surgery	Not reported			.012	13
			Workplace: general hospitals	Not reported			.003	13
			Working more than 10 h per week in clinic	1‐10 h	1.8	1.2‐2.7	<.001	10
			Working more than 10 h per week on bedside	1‐10 h	1.8^a^	1.1‐3.0^a^	.032^a^	10
			Stand more than 75% of the workday	Not reported			.024	13
			Back bending at work	No	8.2 8.3^a^	5.3‐12.9 5.1‐13.4^a^	<.001 <.0001^a^	10
			Pulling objects often at work	No	4.1 3.1^a^	2.4‐7.1 1.7‐5.6^a^	<.001 <.0001^a^	10
			Severe stress level at work	Not reported			.015	13
Dentists	Year	Individual	Female gender	Male	2.17^a^	1.12‐4.20^a^	.021^a^	30
			Increasing age	Continuous	1.068 1.07^a^	1.03‐1.11 1.03‐1.11^a^	<.001 <.001^a^	30
			Lack of exercise	Exercising	2.34	1.25‐4.36	.008	30
			Being married	Not reported			<.001	30
			BMI: obesity	Normal weight	1.72 1.10^a^	1.04‐2.83 1.01‐3.65^a^	<.05 <.05^a^	16
			History of back trauma	No history of back trauma	10.44 11.52^a^	3.79‐28.78 4.14‐32.08^a^	<.05 <.05^a^	16
		Occupational	Specialty: restorative dentists	General practitioner/maxillofacial dentistry	2.82	1.28‐6.21	.019	30
			Specialty: Pediatrics/orthodontics	General practitioner/maxillofacial dentistry	5.21	1.71‐15.83	.019	30
			Specialty: endodontics	General practitioner/maxillofacial dentistry	2.83	1.01‐7.93	.019	30
			Workplace: secondary and tertiary hospitals	Primary	1.80 1.32^a^	1.25‐2.59 1.01‐1.76^a^	<.05 <.05^a^	16
			Experience	Continuous	1.06	1.03‐1.10	.001	30
			Prolonged standing working conditions	Prolonged sitting	1.61	1.01‐2.56	<.05	16
			Increasing time spent per patient	Continuous	1.2	1.00‐1.44	.039	30
			Excessive bending and twisting	Not reported			<.001	30
Nurses	Week	Individual	Nationality: Asian	Western	1.96	1.01‐3.83	.032	9
			BMI: <25	≥30	1.67	1.02‐2.74	.029	9
			Female gender	Not reported			.002	36
		Occupational	Job type: inpatient nurses	Administrative nurses	1.82	1.21‐2.75	.002	9
			Job type: outpatient nurses	Administrative nurses	1.79	1.15‐2.79	.006	9
			Use of patients lifting device	No	1.65	1.21‐2.25	.0008	9
			Carrying patients	No	2.07	1.53‐2.79	<.0005	9
			Supporting patients during movement	No	2.5	1.67‐3.74	<.0005	9
			Pushing wheelchair	No	3.52	2.23‐5.58	<.0005	9
	Year	Individual	Male gender	Females	2.05	1.26‐3.36	.002	9
			Nationality: Middle eastern	Western	2.43	1.02‐5.82	.027	9
			Nationality: Asian	Western	2.24	1.14‐4.38	.011	9
			Age: 30 to <40 y	20 to <30	1.87	1.26‐2.75	<.05	16
			History of back trauma	No history of back trauma	10.44 11.52^a^	3.79‐28.78 4.14‐32.08^a^	<.05 <.05^a^	16
			BMI: obesity	Normal weight	1.72 1.10^a^	1.04‐2.83 1.01‐3.65^a^	<.05 <.05^a^	16
		Occupational	Workplace: secondary and tertiary hospitals	Primary	1.80 1.32^a^	1.25‐2.59 1.01‐1.76^a^	<.05 <.05^a^	16
			Working in surgery department	Not reported	2.2	1‐4.8	<.05	34
			Working in obstetrics & gynecology department	Not reported	1.5	1‐2.1	<.01	34
			Prolonged standing working conditions	Prolonged sitting	1.61	1.01‐2.56	<.05	16
			Handling more than five patients per day	1‐5 patients	1.9^a^	1.15‐3.56^a^	<.05^a^	6
			Increasing time spent in patient handling	Continuous	1.4^a^	1.05‐1.70^a^	<.05^a^	6
			Lack of a workplace patient handling policy	Yes	1.4^a^	1.18‐1.97^a^	<.05^a^	6
	Career	No risk factors identified						
	Lifetime	Individual	Age: <30 y	≥50	4.74	3.06‐7.35	<.0005	9
PTs	Week	No risk factors identified						
	Year	Individual	Female gender	Not reported			.024	15
	Career	No risk factors identified						
EMS	Week	No risk factors identified						
	Year	Individual	Smoking	Not reported			.002	28
			Increasing BMI	Not reported			.009	28
AT	Career	No risk factors identified						
ORT	Career	No risk factors identified						

Abbreviations: AT, anesthesia technicians; BMI, body mass index; CI, confidence interval; EMS, emergency medical service personnel; OR, odds ratio; ORT, operation room technicians; PTs, physical therapists.

^a^ Results are related to adjusted odds ratio.

### 3.7 Sensitivity analysis

High risk of bias was found for eight of the eligible studies. For this reason, they were excluded from the final analysis. A sensitivity analysis was thus performed to explore whether the pooled prevalence rates would change if high risk‐of‐bias studies were included. Based on the prevalence periods identified in this review, the following periods were extracted from the excluded articles as shown in Table S1: week prevalence for nurses (one study),^8^ year prevalence for dentists and medical laboratory technologists (one study each),^22^, ^31^ career prevalence for physicians and nurses (one study each),^14^, ^38^ and lifetime prevalence for physicians (three studies).^14^, ^25^, ^29^ One additional study^26^ reported the career prevalence for dentists, dental assistants, dental hygienists, and dental technicians combined. Therefore, the prevalence estimate reported in this study was not included in the sensitivity analysis.

The sensitivity analysis revealed that recalculating the lifetime prevalence for physicians after including the high risk‐of‐bias studies was comparable to the results when only the studies with acceptable methodology were included; pooled prevalence = 71.0% (95% CI = 59.4%‐82.6%). For career prevalence across all professional groups, the overall career prevalence was 75.1% (95% CI = 64.5%‐86.1%) when the high risk‐of‐bias studies were included in the analysis, which is slightly lower than the prevalence rate calculated with the lower‐of‐bias studies only. Similarly, no considerable differences were noticed in week nor year prevalence rates across all professional groups when estimates from the high risk‐of‐bias studies were included in the meta‐analysis; pooled prevalence = 42.9% (95% CI = 31.2%‐54.6%) and 63.8% (95% CI = 58.9%‐68.8%), respectively. Therefore, excluding the high risk‐of‐bias studies from the meta‐analyses did not have a substantial effect on the LBP prevalence rates calculated based on methodologically superior studies.

## 4 DISCUSSION

The literature search in the present review identified 26 eligible studies examining the prevalence of LBP or musculoskeletal disorders including LBP and the associated risk factors among different groups of health workers in Saudi Arabia. Nearly all of these studies were conducted in the last 10 years. This dramatic increase in the number of studies on that topic in recent years indicates the gravity of the problem and the current interest in investigating the main issues of health workers in relation to the development of LBP. This review provides a comprehensive summary of such attempts in the literature. The information provided in this review is expected to increase awareness in the healthcare sector in about the issue of LBP among health workers in Saudi Arabia.

### 4.1 Prevalence

The meta‐analysis revealed a LBP year prevalence rate of 65.0% for health workers in Saudi Arabia. This is to some degree higher than the rates reported in other cross‐sectional and review studies conducted in the Middle East and internationally. For example, a meta‐analysis conducted in Iran^43^ estimated the year prevalence of LBP among health workers to be 58%. Similarly, other cross‐sectional studies reported an annual prevalence of 39%‐61.3% in Turkey,^44^, ^45^ 51.1% in Tunisia,^46^ 46% in Nigeria,^47^ 56.9% in Malaysia,^48^ and 30% in Ireland.^49^ The week prevalence estimated in the current review was also found to be higher than that estimated for their Turkish counterparts (29.5%).^44^

Nurses are at higher risk of developing LBP than other health workers in Saudi Arabia considering a week period, with an estimated prevalence of 45.9%. In addition, the estimates computed for nurses in the current review were higher than those reported in other reviews. A meta‐analysis of 22 studies in Iran^50^ reported a year prevalence rate among Iranian nurses of 61.2%, which is slightly lower than the rate reported for nurses in this review (66.9%). The worldwide 7‐day and year LBP prevalence rates among nurses were 35% and 55%, respectively,^42^ which are also lower than the pooled week and annual prevalence rates for nurses reported in this review.

For physicians, on the other hand, the prevalence rates calculated in this review are somehow comparable with those reported in another systematic review that included studies from the United States, Ireland, Turkey, Spain, and China.^51^ That review reported a year prevalence ranging from 33% to 68%, and a lifetime prevalence of 67%. The current review found that the pooled year prevalence of LBP among physicians in Saudi Arabia was 66.8%, and the lifetime prevalence was 68.7%. This might indicate a worldwide similarity among physicians in terms of predisposing factors.

The highest year prevalence of LBP was found among dentists, with a pooled rate of 67.3%, which is also higher than the year prevalence of 56.4% reported in Western countries.^52^ Physical therapists, among other health workers in Saudi Arabia, showed the highest risk of developing LBP over their career, with a prevalence rate of 89.6%. This might not be surprising, since physical therapists routinely perform manual therapy techniques and repetitive tasks that sometimes involve heavy physical demands.^53^ A previous study found that physical therapists were more vulnerable to work‐related musculoskeletal disorders during their career than other health workers.^54^ The results of this review confirm this finding.

It should be mentioned that the lifetime prevalence described in this review must be inferred with caution, as it represents physicians only. This might also explain the higher rate reported for career prevalence, as this rate was computed based on data taken from different categories (physicians, nurses, physical therapists, ORT, and AT).

In some instances, the prevalence rates for the same occupational category showed some variability between the studies. This was mainly evident in the year prevalence for dentists. Perhaps the lack of a uniform case definition of LBP might explain this variability. Although half of the studies standardized the definition of LBP utilizing the Standardized Nordic Musculoskeletal Questionnaire, and one study utilized the Cornell Musculoskeletal Discomfort Questionnaire, the remaining studies recorded and defined LBP incidents using self‐designed questionnaires. However, this high variability in prevalence rates was not found in the year prevalence among nurses, which was reported in five studies and ranged between 63.8%^23^ and 74.8%,^6^ regardless of the LBP definition choice. This indicates that other factors might lead to this variability as well, such as the setting where the study was conducted. Two studies in the current analysis concluded that the prevalence of LBP differed based on type of healthcare facility.^13^, ^16^ Another factor could be the specialty, as some studies found significant differences in LBP prevalence among physicians and dentists based on their specialty.^10^, ^13^, ^30^, ^32^

Another observed variability between the 18 studies that were included in the analyses (due to having low or medium risk of bias) was in the eligibility criteria for LBP type (specific vs nonspecific). Although four studies^13^, ^15^, ^32^, ^36^ were clear about only including cases with nonspecific LBP by excluding those with LBP secondary to other pathology or abnormality, the rest did not state precise eligibility criteria related to the type of LBP. At the same time, all 18 studies defined LBP as “work‐related LBP” and/or attributed it to work‐related factors, and none was linked to disease. This inconsistency made it difficult to classify cases into specific and nonspecific, and may have contributed to the variability in prevalence rates between the studies.

### 4.2 Duration and intensity of LBP episodes

Estimating and comparing the duration and intensity of LBP episodes was challenging in this review for two reasons. First, only five^6^, ^7^, ^30^, ^32^, ^36^ out of the 18 studies reported the duration, and eight articles^7^, ^10^, ^13^, ^30^, ^32^, ^35^, ^36^, ^37^ provided data about the intensity. This may provide insufficient estimates about the actual duration and intensity of LBP. Second, studies lacked standardized methods for reporting duration and intensity. This inconsistency makes comparisons among occupational categories difficult.

Overall, duration of LBP episodes was reported for physicians, dentists, nurses, and physical therapists, with approximately 80%‐86% describing their pain as acute (less than 4 weeks). Intensity can be described as moderate for physicians, dentists, nurses, physical therapists, AT, and ORT based on the data reported. However, these inferences must be made with caution, as further studies are needed to provide adequate estimates and comparisons of the duration and intensity of LBP episodes among health workers in Saudi Arabia.

### 4.3 Risk factors

#### 4.3.1 Individual risk factors

The analysis of individual risk factors of LBP in health workers in Saudi Arabia revealed that as age and BMI increased, so did the likelihood of developing LBP. However, one study^9^ reported a higher prevalence of LBP among participants with lower BMI and younger age. A similar conflict was found for gender, as three studies^15^, ^30^, ^36^ reported that female gender was associated with higher LBP prevalence, while two studies^9^, ^10^ reported that male gender was a significant risk factor of LBP. Nevertheless, increasing age and weight, and female gender are well‐documented risk factors of LBP in the literature.^55^ Other relatively common risk factors reported were smoking and nationality, with non‐Saudi health workers being more vulnerable to developing LBP. One study hypothesized that this might be because the Saudi participants in their study were younger than non‐Saudis.^13^ Another possible explanation could be that the contracts of non‐Saudi staff are renewed every year based on their performance,^56^ and job insecurity was found to be significantly associated with LBP,^57^ which may also explain this finding.

#### 4.3.2 Occupational risk factors

The majority of occupational risk factors were related to the type of activities performed at work with high physical demands, including those requiring bending and twisting, and lifting and pulling objects. Alsiddiky et al^10^ reported that clinicians who often performed back bending and pulling objects at work had a risk of LBP up to eight times higher. Back flexion, especially when combined with lifting weights, has serious consequences on the lower back, as it highly increases the intradiscal pressure,^58^ and may damage the discs. Similarly, work activities involving patient manual‐handling, mainly among nurses, were also identified as risk factors, such as transferring and carrying patients, supporting patients during movement, pushing wheelchairs, increased time spent handling patients, and number of patients handled. The highest risk of LBP was found among nurses who often pushed wheelchairs (three times higher).^9^ These findings are in agreement with previous reviews conducted internationally.^57^, ^59^, ^60^ Some explanations of the relationship between these types of activities and LBP in nurses were reported, such as a reduction in the ability to endure the physical load among those with weak muscle strength^61^ or lack of knowledge about ergonomically safe patient‐handling techniques.^45^ Organizational factors may also play a role, as Al‐Eisa and Al‐Abbad^6^ concluded that the absence of a workplace patient handling policy was a significant risk factor for LBP in nurses. One study,^9^ however, reported that the utilization of patient‐lifting devices does not protect nurses against LBP, as it was found to be positively correlated with LBP occurrence. Nevertheless, it was previously reported that it might take up to 4 years of follow‐up to detect the effect of those devices on reducing the LBP incidence.^60^ Moreover, the beneficial effect of implementing patient‐lifting devices on LBP and musculoskeletal disorders among health workers is well documented in the literature among newly recruited staff^60^ and when combined with other preventive strategies.^62^

Working department and workplace were also recognized as risk factors of LBP. Those who worked in hospitals (secondary, tertiary, or general hospitals) were at higher risk of LBP than their counterparts who worked in small or primary health centers, which is consistent with a previous review.^57^ This is possibly due to extended working hours and higher patient loads associated with stressful working environments.^63^ Furthermore, nurses who worked in surgical departments were found to be twice more likely to suffer from LBP than those in other departments,^35^ which is in agreement with an earlier report.^64^ Similarly, inpatient and outpatients nurses, as compared with administrative nurses, were at a higher risk of LBP.^9^ Variations in workplace equipment^11^, ^60^, ^65^ and work systems and duties^6^, ^44^, ^48^, ^66^ could explain these findings.

Certain subspecialties among physicians and dentists were also noticed to be more susceptible to LBP. Among dentists, pediatric dentists, orthodontists, restorative dentists, and endodontic dentists were found to be at a higher risk of LBP in comparison with general dentists and maxillofacial surgeons. A greater risk was found for orthodontics and pediatrics dentist (five times higher), followed by endodontics and restorative dentists (approximately three times higher).^30^ Maintaining an awkward static posture for extended periods of time is the most commonly reported explanation for such high risk of LBP among different dental specialties.^30^, ^67^ Similarly, among physicians, orthopedic and general surgeons, gynecologists, pediatricians, ophthalmologists, emergency and intensive care physicians, and anesthesiologists were at a greater risk of LBP development than other specialties, which can be explained by extended procedure times and high physical and mental demands in those specialties.^13^, ^30^

High stress level at work is a well‐documented risk factor of LBP,^57^, ^63^ and its negative impact on work performance among health workers has been established.^68^ However, two studies included in the review examined stress, and only one found it to be significant.^13^ Earlier reports found that prolonged standing induces LBP. This was explained by multiple reasons such as standing in more lumbar lordosis^69^ and alteration of muscle activities around the back.^70^ In this review, moreover, it was found that those who spent most of their time working in a standing position were around 1.5 time more likely to develop LBP.^16^ However, it has been suggested that sitting breaks alone do not protect from the harmful effect of prolonged standing on the lower back region, and those periods of rest should include other types of activities.^71^ Years of experience was also found to have a significant association with LBP. This could be a risk factor because of its direct proportionality with age, which was identified as a significant risk factor, as described earlier.

### 4.4 Recommendations for occupational health

This review identified a number physical work‐related factors associated with LBP occurrence, ranging from maintenance of static posture combined with excessive back bending and twisting among dentists, to manual patient‐handling and repetitive heavy lifting among nurses. Possibly, a midpoint between the two ends of physical demands could help to minimize the risk of LBP in these two categories. For example, dentists could take more frequent breaks that include walking around and performing some stretching exercises.^30^ Nurses, on the other hand, would be recommended to take a rest from heavy physical workloads on a regular basis.^6^ Modifying the workplace,^30^, ^33^ implementing safety polices at work,^6^ revising the working hours,^10^ recruiting enough staff,^6^, ^16^ and increasing awareness about safe ergonomics at work^6^, ^15^ are recommended for both groups in addition to other health workers. Additionally, several studies included in this analysis documented the benefits of exercising as a protective factor against LBP.^6^, ^16^, ^30^ Occupational health workers are recommended to spread the knowledge about the beneficial effects of regular exercise in minimizing the risk of LBP occurrence. The prophylactic measures suggested here are provided for the healthcare sector in Saudi Arabia and potentially in other countries, since there are universal similarities in the predisposing factors of LBP.

### 4.5 Study limitations

A potential limitation of this review is the occupational categories covered. Although the search criteria included all possible health professionals and/or health associate professionals, only seven categories were identified and included. Another limitation is that the representation of the occupations included in the analysis was not equal. This was due to a general lack of studies targeting some categories. A third limitation is that some regions of Saudi Arabia were not covered because of the lack of studies in those regions. A fourth limitation is the inconsistency of the eligibility criteria among the included studies in terms of type of LBP, which made it difficult to categorize the cases as specific and nonspecific LBP. Furthermore, the case definition of LBP in the studies included in the analysis was not consistent, and the duration and intensity of LBP episodes were not recorded by all studies. These limitations may have influenced the prevalence rate estimates reported in this review. A fifth limitation is that only one reviewer performed the selection of the studies, and only English articles were considered for eligibility in this review. Finally, as only cross‐sectional studies were included, causal relationships between LBP and the identified risk factors cannot be established.

### 4.6 Recommendations for future research

Based on the results of the present review and to further improve the overall understanding of LBP prevalence in the healthcare sector in Saudi Arabia, the following recommendations are provided: (a) future studies need to examine the frequency and risk factors of LBP among other common health workers such as pharmacists, medical laboratory technologists, and other allied health workers; (b) future studies need to be conducted in other regions of Saudi Arabia such as Northern Borders, Jawf, Al‐Madinah, Al‐Bahah, Jazan, Najran, and Al‐Qassim; (c) future studies need to report the duration and intensity of LBP episodes using a consistent method, and to use a uniform, standardized case definition of LBP, such as the Standardized Delphi Definitions of Low Back Pain Prevalence,^72^ to facilitate the comparison of prevalence rates between different groups; and (d) future studies need to include precise criteria regarding the type of LBP (specific vs nonspecific), as such data would add important information on the type of LBP in relation to prevalence rates.

## 5 CONCLUSIONS

Compared with the rest of the world, LBP is highly prevalent among health workers in Saudi Arabia, with rates of 40.8%, 65.0%, and 81.4% for week, year, and career prevalence, respectively. Nurses were more susceptible to LBP over a 7‐day period, while physical therapists were more likely to develop LBP over their career. Occupational risk factors were mostly related to work‐related activities and workplace facilities. To limit LBP and minimize its consequences, working policies in the Saudi healthcare sector might need to be reviewed, and proper protective measures need to be developed. Moreover, enough staff need to be recruited to reduce the patient‐to‐staff ratio and working hours and thus decrease the workload. Work organizations need to consider adopting prophylactic strategies, including redesigning the workplace, adequately implementing lifting devices, and appropriate education and training of staff about correct patient handling techniques, safe ergonomics and body mechanics, and health benefits of exercising. Such modifications would help to reduce the incidence of LBP and associated disabilities among health workers in Saudi Arabia. This, in turn, would improve the quality of patient care by keeping health staff active and productive during their career.

## DISCLOSURE

*Approval of the research protocol*: N/A *Informed consent*: N/A *Registry and the registration no. of the study/trial*: N/A *Animal studies*: N/A *Conflict of Interest*: Authors declare no conflict of interests for this article.

## AUTHOR CONTRIBUTIONS

Hamad S. Al Amer performed the literature search, data analyses, and wrote and revised the manuscript.

## Supporting information

Fig S1‐S9

Table S1
