# Supplementary material for: Low back pain prevalence and risk factors among health workers in Saudi Arabia: A systematic review and meta‐analysis
Source: J Occup Health. 2020 Jul 25;62(1):e12155. doi: 10.1002/1348-9585.12155 (PMC7382437; doi:10.1002/1348-9585.12155)
Supplement: Supplementary file 1 — Fig S1‐S9 [file JOH2-62-e12155-s001.pdf]

Supplemental Figure 1. Week prevalence of low back pain among health professionals in Saudi Arabia

Study name	Subgroup	Event rate	Lower limit	Upper limit
**Alzidani et al. 2018[13]**	**Physicians**	**0.203**	**0.128**	**0.278**
**Al Shammari et al. 2019[24]**	**Physicians**	**0.424**	**0.334**	**0.515**
**Mohamed and Al Amer 2019[37]**	**Physicians**	**0.500**	**0.308**	**0.692**
**Abbas et al. 2010[9]**	**Nurses**	**0.615**	**0.565**	**0.665**
**Abu Tariah et al. 2020[23]**	**Nurses**	**0.170**	**0.087**	**0.254**
**Keriri 2013[36]**	**Nurses**	**0.484**	**0.363**	**0.606**
**Mohamed and Al Amer 2019[37]**	**Nurses**	**0.575**	**0.409**	**0.741**
**Mohamed and Al Amer 2019[37]**	**PT**	**0.357**	**0.136**	**0.579**
**Aljerian et al. 2018[28]**	**EMS**	**0.372**	**0.309**	**0.435**
		**0.408**	**0.284**	**0.532**

Random-effects Model (I^2 = 93.9%)

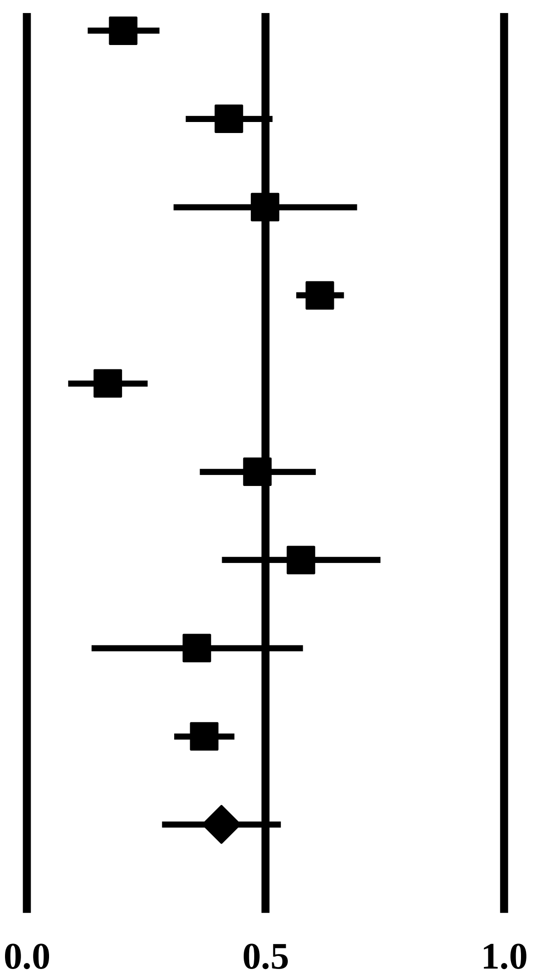

Abbreviations: CI, confidence interval; PT, physical therapists; EMS, emergency medical service.

Supplemental Figure 2. Year prevalence of low back pain among health professionals in Saudi Arabia

Study name	Subgroup	Event rate	Lower limit	Upper limit
Alzidani et al. 2018[13]	Physicians	0.659	0.524	0.795
Al Shammari et al. 2019[24]	Physicians	0.692	0.576	0.808
Alnaami et al. 2019[16]	Physicians	0.734	0.644	0.823
Al-Ruwaili and Khalil 2019[32]	Physicians	0.697	0.594	0.800
Alsultan et al. 2018[33]	Physicians	0.529	0.408	0.649
Aljanakh et al. 2015[27]	Dentists	0.574	0.394	0.754
Al-Mohrej et al. 2016[30]	Dentists	0.681	0.568	0.795
Alnaami et al. 2019[16]	Dentists	0.926	0.563	1.289
Al-Eisa and Al-Abbad 2013[6]	Nurses	0.748	0.612	0.885
Abbas et al. 2010[9]	Nurses	0.652	0.600	0.704
Abu Tariah et al. 2020[23]	Nurses	0.638	0.477	0.800
Alnaami et al. 2019[16]	Nurses	0.729	0.615	0.843
Attar 2014[34]	Nurses	0.650	0.538	0.762
Muaidi and Shanb 2016[15]	PT	0.465	0.414	0.516
Aljerian et al. 2018[28]	EMS	0.603	0.523	0.683
		0.650	0.594	0.705

Random-effects Model (I^2 = 77.1%)

Event rate and 95% CI

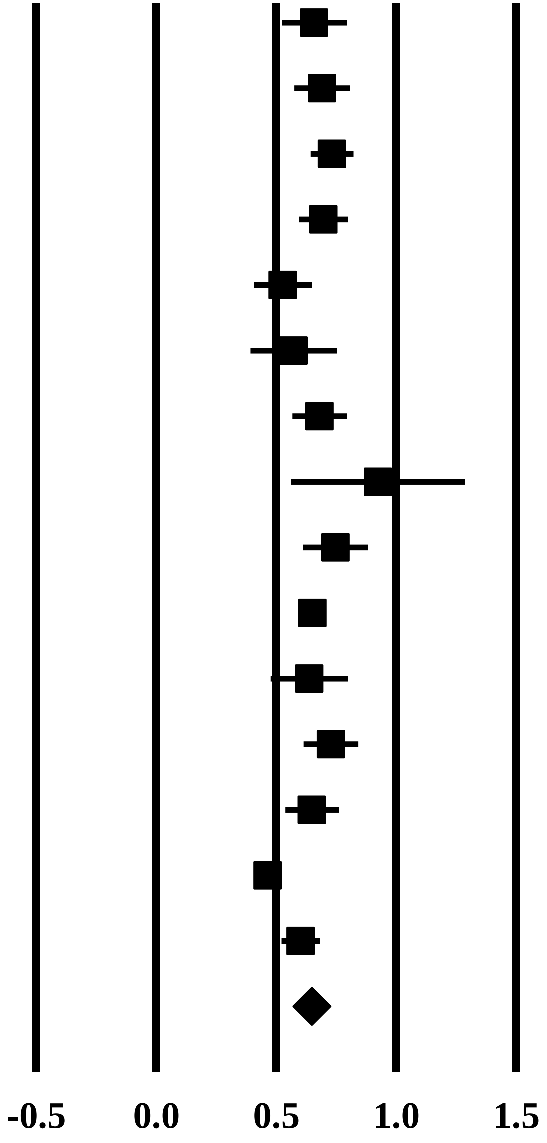

Abbreviations: CI, confidence interval; PT, physical therapists; EMS, emergency medical service.

Supplemental Figure 3. Career prevalence of low back pain among health professionals in Saudi Arabia

Study name	Subgroup	Event rate	Lower limit	Upper limit
**Bin Homaid et al. 2016[35]**	**Physicians**	**0.738**	**0.522**	**0.953**
**Bin Homaid et al. 2016[35]**	**Nurses**	**0.765**	**0.471**	**1.059**
**Alghadir et al. 2017[7]**	**PT**	**0.896**	**0.814**	**0.979**
**Bin Homaid et al. 2016[35]**	**AT**	**0.833**	**0.317**	**1.350**
**Bin Homaid et al. 2016[35]**	**ORT**	**0.429**	**-0.056**	**0.914**
		**0.814**	**0.693**	**0.935**

Random-effect Model (I^2 = 26.3%)

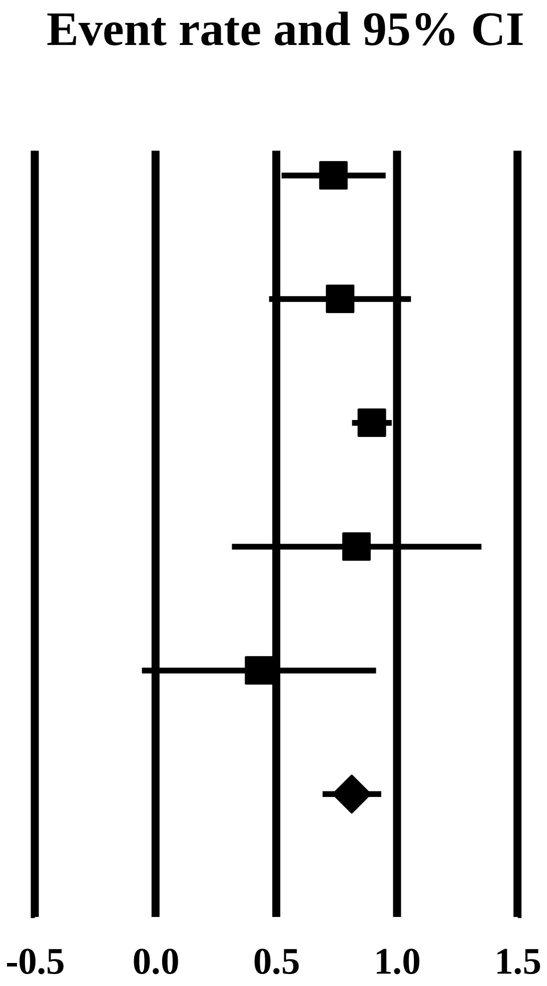

Abbreviations: CI, confidence interval; PT, physical therapists; AT, anesthesia technicians; ORT, operation room technicians.

Supplemental Figure 4. Week prevalence of low back pain among physicians in Saudi Arabia

Study name	Event rate	Lower limit	Upper limit
**Alzidani et al. 2018[13]**	**0.203**	**0.128**	**0.278**
**Al Shammari et al. 2019[24]**	**0.424**	**0.334**	**0.515**
**Mohamed and Al Amer 2019[37]**	**0.500**	**0.308**	**0.692**
	**0.364**	**0.180**	**0.547**

Random-effects Model (I^2 = 88.6%)

Event rate and 95% CI

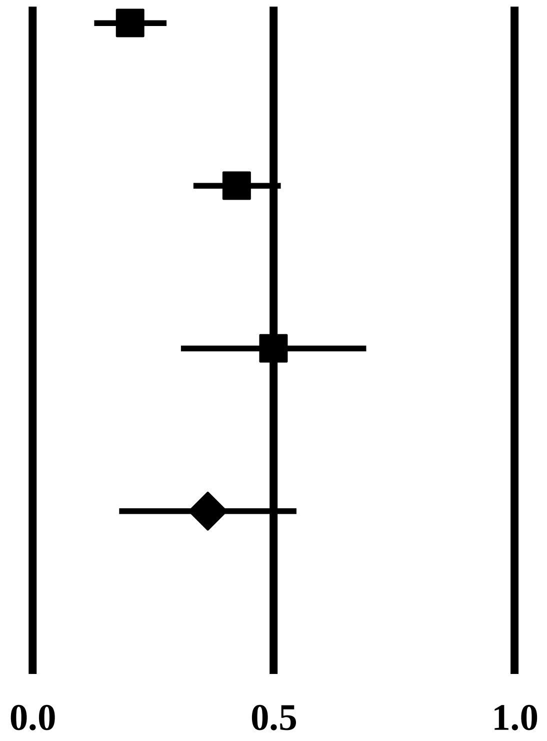

Abbreviations: CI, confidence interval.

Supplemental Figure 5. Year prevalence of low back pain among physicians in Saudi Arabia

Study name	Event rate	Lower limit	Upper limit
**Alzidani et al. 2018[13]**	**0.659**	**0.524**	**0.795**
**Al Shammari et al. 2019[24]**	**0.692**	**0.576**	**0.808**
**Alnaami et al. 2019[16]**	**0.734**	**0.644**	**0.823**
**Al-Ruwaili and Khalil 2019[32]**	**0.697**	**0.594**	**0.800**
**Alsultan et al. 2018[33]**	**0.529**	**0.408**	**0.649**
	**0.668**	**0.599**	**0.737**

Random-effect Model (I^2 = 47.6%)

Event rate and 95% CI

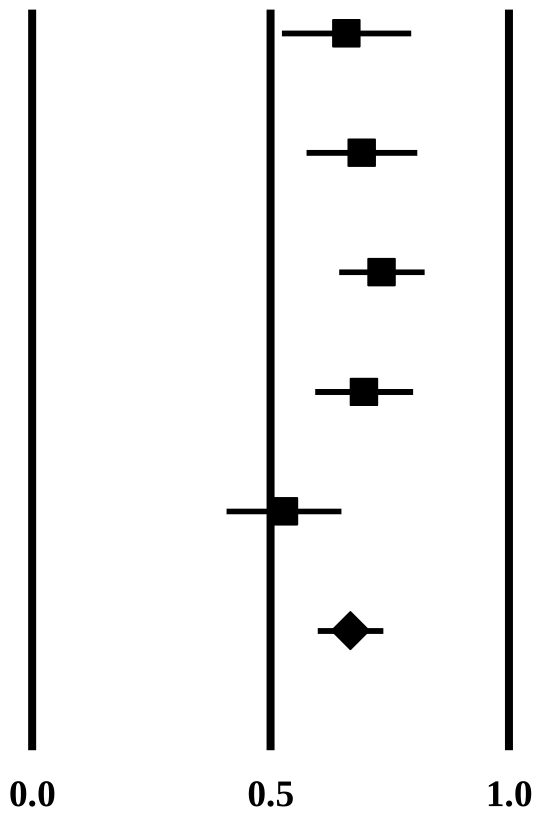

Abbreviations: CI, confidence interval.

Supplemental Figure 6. Lifetime prevalence of low back pain among physicians in Saudi Arabia

Study name	Event rate	Lower limit	Upper limit
**Alsiddiky et al. 2015[10]**	**0.594**	**0.519**	**0.668**
**Alzidani et al. 2018[13]**	**0.732**	**0.589**	**0.875**
**Al-Ruwaili and Khalil 2019[32]**	**0.760**	**0.653**	**0.867**
**Random-effects Model (I^2 = 72.8%)**	**0.687**	**0.570**	**0.804**

Event rate and 95% CI

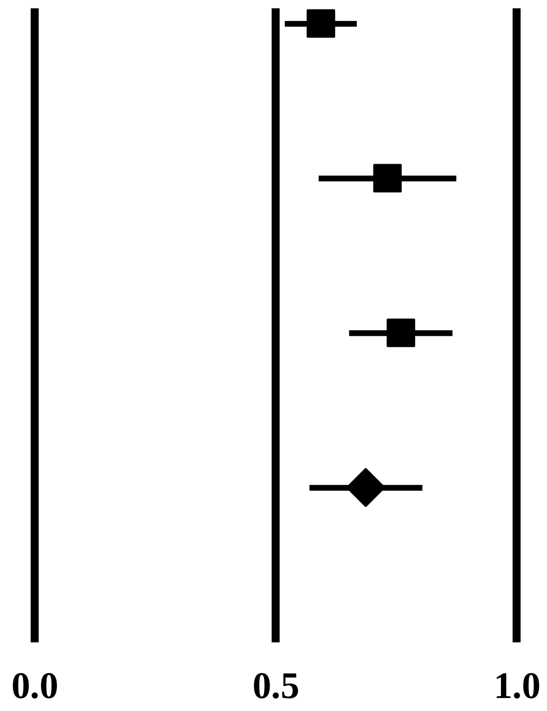

Abbreviations: CI, confidence interval.

Supplemental Figure 7. Year prevalence of low back pain among dentists in Saudi Arabia

Study name	Event rate	Lower limit	Upper limit
**Aljanakh et al. 2015[27]**	**0.574**	**0.394**	**0.754**
**Al-Mohrej et al. 2016[30]**	**0.681**	**0.568**	**0.795**
**Alnaami et al. 2019[16]**	**0.926**	**0.563**	**1.289**
**Random-effects Model (I^2 = 34.4%)**	**0.673**	**0.541**	**0.805**

Event rate and 95% CI

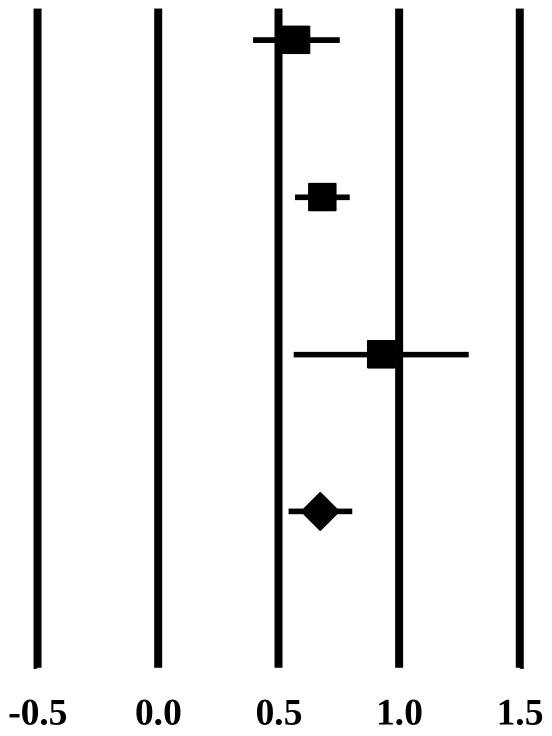

Abbreviations: CI, confidence interval.

Supplemental Figure 8. Week prevalence of low back pain among nurses in Saudi Arabia

Study name	Event rate	Lower limit	Upper limit
**Abbas et al. 2010[9]**	**0.615**	**0.565**	**0.665**
**Abu Tariah et al. 2020[23]**	**0.170**	**0.087**	**0.254**
**Keriri 2013[36]**	**0.484**	**0.363**	**0.606**
**Mohamed and Al Amer 2019[37]**	**0.575**	**0.409**	**0.741**
	**0.459**	**0.219**	**0.699**

Random-effect Model (I^2 = 96.3%)

Event rate and 95% CI

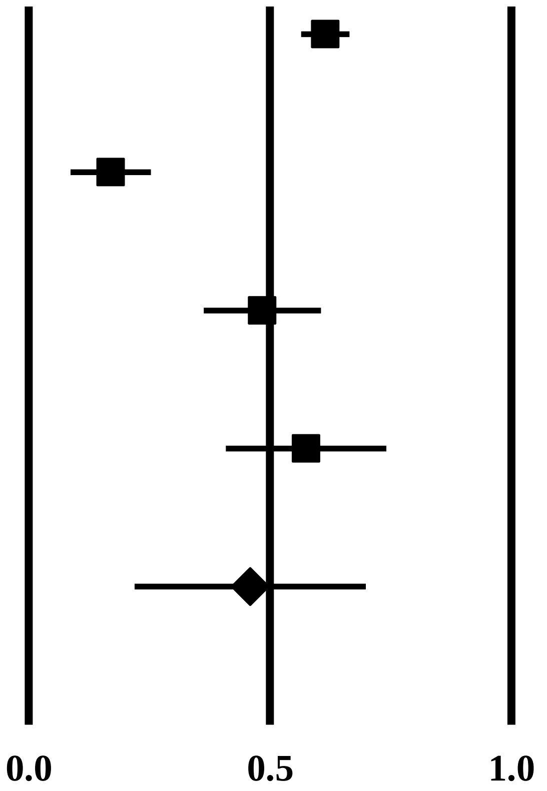

Abbreviations: CI, confidence interval.

Supplemental Figure 9. Year prevalence of low back pain among nurses in Saudi Arabia

Study name	Event rate	Lower limit	Upper limit
**Al-Eisa and Al-Abbad 2013[6]**	**0.748**	**0.612**	**0.885**
**Abbas et al. 2010[9]**	**0.652**	**0.600**	**0.704**
**Abu Tariah et al. 2020[23]**	**0.638**	**0.477**	**0.800**
**Alnaami et al. 2019[16]**	**0.729**	**0.615**	**0.843**
**Attar 2014[34]**	**0.650**	**0.538**	**0.762**
	**0.669**	**0.629**	**0.709**

Fixed-effect Model (I^2 = 0%)

Event rate and 95% CI

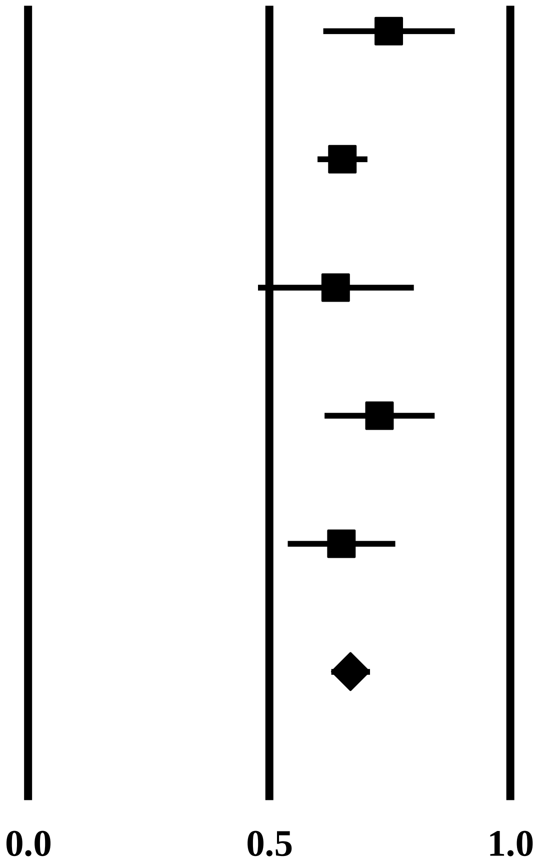

Abbreviations: CI, confidence interval.
