# Supplementary material for: Low back pain prevalence and risk factors among health workers in Saudi Arabia: A systematic review and meta‐analysis
Source: J Occup Health. 2020 Jul 25;62(1):e12155. doi: 10.1002/1348-9585.12155 (PMC7382437; doi:10.1002/1348-9585.12155)
Supplement: Supplementary file 2 — Table S1 [file JOH2-62-e12155-s002.pdf]

Supplemental Table 1. Prevalence rates of low back pain from studies with high risk of bias

Study	Occupation	Prevalence period and rate			
		**Week**	**Year**	**Career**	**Lifetime**
Al Dajah and Al Daghdi 2013[8]	Nurses	61.3%	-	-	-
Aseri et al. 2019[14]	Physicians	-	-	69%	84.9%
Abduljabbar 2008[22]	Dentists	-	52.1%	-	-
Alamri et al. 2018[25]	Physicians	-	-	-	47.8%
Alghadir et al. 2015[26]	Dental professionals[a]	-	-	50.6%	-
Almalki et al. 2016[29]	Physicians	-	-	-	87.7%
AlNekhilan et al. 2020[31]	MLT	-	60.9%	-	-
Gaowgzeh 2019[38]	Nurses	-	-	66.7%	-

[a]Include dentists, dental assistants, dental hygienists, and dental technicians.

Abbreviations: MLT, medical laboratory technologists.
